# Antimitochondrial antibody associated with liver cirrhosis in patients with primary biliary cholangitis

**DOI:** 10.1097/MD.0000000000035617

**Published:** 2023-10-20

**Authors:** Yujiao Jin, Miaochan Wang, Yuan Liu, Aifang Xu

**Affiliations:** a Department of Clinical Laboratory, Xixi Hospital of Hangzhou, Hangzhou, Zhejiang, China.

**Keywords:** antimitochondrial antibodies, cirrhosis, primary biliary cholangitis

## Abstract

Antimitochondrial antibody (AMA) serves as a serological marker for diagnosing primary biliary cholangitis (PBC). However, the association between AMA and prognosis for PBC patients remains unclear. The objective of this study was to investigate the relationship between AMA and cirrhosis in PBC patients. This retrospective study enrolled 225 PBC patients, including 127 with liver cirrhosis and 98 without cirrhosis. AMA was tested by indirect immunofluorescence (IIF) with rat kidney as the substrate. AMA-M2 and M2-3E were detected by line immunoassay (LIA). The overall positivity rate for AMA detection in PBC patients was 80.9%. The positivity rates of IIF-AMA, AMA-M2, and M2-3E were significantly higher in patients with liver cirrhosis than in those without cirrhosis (73.2% vs. 52.0%, 74.0% vs. 51.0%, and 80.3% vs. 60.2%, respectively). In multivariate logistic regression, IIF-AMA (OR: 3.05, 95% CI: 1.59–5.87), AMA-M2 (OR: 3.11, 95% CI: 1.61–6.01), and M2-3E (OR: 3.29, 95% CI: 1.63–6.66) remained significantly associated with an increased incidence of liver cirrhosis. Moreover, in multinomial logistic regression, IIF-AMA (compensated cirrhosis, OR: 3.55, 95% CI: 1.49–8.44; decompensated cirrhosis, OR: 2.86, 95% CI: 1.32–6.18), AMA-M2 (compensated cirrhosis, OR: 4.74, 95% CI: 1.94–11.58; decompensated cirrhosis, OR: 2.51, 95% CI: 1.19–5.33), and M2-3E (compensated cirrhosis, OR: 4.92, 95% CI: 1.74–13.96; decompensated cirrhosis, OR: 2.91, 95% CI: 1.28–6.64) were all found to be associated with different stages of liver cirrhosis. AMA was found to be associated with the occurrence of liver cirrhosis in PBC patients. Additionally, AMA was also related to different stages of liver cirrhosis, including compensated and decompensated cirrhosis.

## 1. Introduction

Primary biliary cholangitis (PBC), formally known as primary biliary cirrhosis, is a chronic cholestatic liver disease that mainly affects middle-aged women.^[1]^ PBC, considered as an autoimmune liver disease, is characterized by cholestasis, the presence of disease-specific autoantibodies including antimitochondrial antibodies, and histologic evidence of chronic nonsuppurative destructive cholangitis affecting small- or medium-size bile ducts.^[2]^ The disease is often progressive, eventually resulting in liver cirrhosis and liver failure without appropriate treatment.^[3]^

AMA, the hallmark of PBC, can be detected in approximately 90% of patients with PBC.^[2]^ AMA recognizes the 2-oxo-acid dehydrogenase complex located in the inner membrane of the mitochondria, which mainly comprises the pyruvate dehydrogenase complex E2 subunit (PDC-E2), the 2-oxo-glutaric acid dehydrogenase complex E2 subunit (OGDC-E2), and the branched-chain 2-OADC E2 subunit (BCOADC-E2).^[4]^ As the most disease-specific autoantibodies, AMA serves as a serological marker for diagnosing PBC. Additionally, highly specific antinuclear antibodies (ANA) for PBC, such as Sp100 and gp210 autoantibodies, can also aid in the diagnosis of PBC, especially in AMA-negative patients.^[5]^ In particular, the presence of anti-gp210 antibodies may be associated with disease progression in PBC patients.^[6]^ However, AMA titers are not typically reported to correlate with progression in patients with PBC.^[7]^ Nevertheless, accumulating evidence suggests that AMA titers may be associated with the outcome of PBC patients.^[8–10]^

Currently, there is still controversy regarding the relationship between AMA and prognosis for PBC patients. Accordingly, we conducted a retrospective study to investigate the association between AMA and liver cirrhosis in PBC patients and to establish the relationship between AMA and different stages of liver cirrhosis.

## 2. Materials and methods

### 2.1. Patients and study design

This retrospective study continuously recruited patients with PBC who were over 18 years old, admitted to Xixi Hospital of Hangzhou, and underwent AMA testing between January 2020 and January 2023. PBC was diagnosed according to the AASLD guidelines, which require meeting at least 2 of 3 criteria: alkaline phosphatase (ALP) elevation, presence of AMA or other PBC-specific antibodies (including Sp100 or gp210), and liver histology compatible with PBC.^[11]^ All enrolled PBC patients were administered ursodeoxycholic acid (UDCA) at a regular dosage of 13 to 15 mg/kg/day. Subjects with comorbid liver diseases, including hepatitis B virus infection, autoimmune hepatitis, hepatitis C virus infection, drug-related hepatitis, and alcohol hepatitis, were excluded. Patients with PBC were divided into cirrhotic and non-cirrhotic groups based on whether they had cirrhosis or not. Liver cirrhosis was diagnosed according to the Chinese guidelines on the management of liver cirrhosis.^[12]^ A diagnosis of liver cirrhosis was based on a comprehensive evaluation of medical history, clinical manifestations, laboratory tests, imaging studies, and histological examinations. Decompensated cirrhosis was diagnosed based on the presence of cirrhosis and any of the following complications: ascites, sepsis, hemorrhage due to gastroesophageal varices, hepatic encephalopathy, or hepatorenal syndrome.^[12]^

The Ethics Committee of Xixi Hospital of Hangzhou approved this study (2023048). Written informed consent was waived due to the retrospective nature of the study.

### 2.2. Clinical information

All relevant medical data, including general information and biochemical characteristics, were gathered from electronic medical records. General information included age, sex, and comorbidities. Biochemistry tests included albumin (ALB), alanine aminotransferase (ALT), aspartate aminotransferase (AST), total bilirubin (T.Bil), ALP, creatinine, platelet count (PLT), prothrombin time (PT), and international normalized ratio (INR). The biochemical characteristics were selected from the most recent day after first admission to the hospital and detected using customary automated methods.

### 2.3. Autoantibodies detection

AMA was detected using the Euroimmun test kit (EUROIMMUN, Germany) by indirect immunofluorescence (IIF), with rat kidney as the substrate. Sera were considered positive for AMA if there was a positive reaction at a dilution of 1:100, as stated in the manual. Two independent evaluators assessed positivity and patterns using fluorescence microscopy. PBC-related autoantibodies, including AMA-M2 (pyruvate dehydrogenase complex), M2-3E (BPO, fusion protein of the E2 subunits of the alpha-2-oxoacid dehydrogenases of the inner mitochondrial membrane), Sp100 (nuclear granula protein, nuclear dots), and gp210 (integral protein of the nuclear membrane, nuclear pore complex) antibodies, were detected using the Euroimmune Test System (EUROIMMUN, Germany) by line immunoassay (LIA). The EUROLINE test kit provides qualitative testing of human autoantibodies. According to the manufacturer instructions, assays were performed, and results were interpreted based on signal intensity using EUROLine Scan software. Results were classified as follows: negative, no signal (0–5); positive, very weak to strong signal (6–50); strong positive, very strong signal (>50).

### 2.4. Statistical analysis

Continuous normally distributed variables were expressed as mean ± SD and compared using the Student *t* test. Non-normally distributed data were expressed as medians with first and third quartiles and compared using the Mann–Whitney test. Categorical variables were expressed as percentages and compared using the χ^2^ test or Fisher exact test as necessary. To exclude the influence of possible confounders on the association of autoantibodies and cirrhosis, a logistic regression model was used. The agreement between AMA detection methods was evaluated using the Kappa coefficient, where values of 0 to 0.2 indicate slight agreement, 0.2 to 0.4 indicate fair agreement, 0.4 to 0.6 indicate moderate agreement, 0.6 to 0.8 indicate substantial agreement, and >0.8 indicate excellent agreement. All statistical analyses were conducted using IBM SPSS Statistics version 24. A 2-tailed *P* value of <.05 was considered statistically significant.

## 3. Results

A total of 254 consecutive patients with PBC were recruited for the present research. Of these, 29 patients were excluded due to comorbid other liver disease. The remaining 225 patients with PBC were enrolled, including 127 with liver cirrhosis and 98 without cirrhosis. Among the cirrhosis patients, 82 were in the decompensated stage. Several AMA detection methods were used in our study, including IIF-AMA, AMA-M2 (LIA), and M2-3E (LIA). The overall positivity rate for AMA detection in PBC patients was 80.9%. Among the AMA negative patients, the positivity rates for Sp100 and gp210 were 23.3% and 25.6%, respectively.

Table 1 shows the baseline characteristics of patients with PBC. The mean age of the patients included was 57.0 ± 11.1 years, and 28 patients (12.4%) were male. Patients with liver cirrhosis were significantly older than those without cirrhosis (60.2 ± 9.4 vs. 53.2 ± 11.1 years). Among the patients with PBC, 43 (19.1%) had hypertension, 34 (15.1%) had diabetes, and 10 (4.4%) had hyperlipidemia. Liver cirrhosis patients had significantly higher levels of AST, T.Bil, ALP, creatinine, and INR and lower levels of ALB and PLT than non-cirrhosis patients. However, no significant differences were observed in ALT and PT. The positivity rates of IIF-AMA, AMA-M2, and M2-3E were significantly higher in liver cirrhosis patients than in non-cirrhosis patients (73.2% vs. 52.0%, 74.0% vs. 51.0%, and 80.3% vs. 60.2%, respectively). The positivity rate of gp210 was also higher in cirrhosis patients than in non-cirrhosis patients (38.6% vs. 25.5%). However, there was no significant difference in Sp100 between the 2 groups.

**Table 1:** Comparison of baseline characteristics between cirrhosis and non-cirrhosis patients.

Characteristic	Total (n = 225)	Cirrhosis (n = 127)	Non-cirrhosis (n = 98)	*P*
Age (yr), mean ± SD	57.0 ± 11.1	60.2 ± 9.4	53.2 ± 11.1	<.001
Gender (male) [n (%)]	28 (12.4)	16 (12.6)	12 (12.2)	.937
Comorbidities [n (%)]				
Hypertension	43 (19.1)	23 (18.1)	20 (20.4)	.664
Diabetes	34 (15.1)	23 (18.1)	11 (11.2)	.153
Hyperlipidemia	10 (4.4)	7 (5.5)	3 (3.1)	.577
Laboratory indexes				
Albumin (g/L), mean ± SD	37.2 ± 9.0	33.5 ± 9.3	41.9 ± 5.8	<.001
Alanine aminotransferase (U/L) [median (IQR)]	30.0 (19.0–45.0)	28.0 (19.0–44.0)	30.0 (18.0–50.0)	.470
Aspartate aminotransferase (U/L) [median (IQR)]	41.0 (28.5–65.5)	46.0 (33.0–73.0)	34.5 (25.0–49.0)	.001
Total bilirubin (μmol/L) [median (IQR)]	16.2 (11.7–22.3)	18.2 (12.9–35.0)	13.9 (11.4–18.4)	<.001
Alkaline phosphatase (U/L) [median (IQR)]	143.0 (96.5–219.0)	148.0 (103.0–241.5)	126.0 (85.3–198.3)	.030
Creatinine (μmol/L) [median (IQR)]	59.0 (50.0–69.0)	61.0 (52.0–70.8)	58.0 (48.3–65.8)	.040
Platelet count (10^9^/L) [median (IQR)]	147.0 (68.5–200.0)	81.0 (49.0–153.0)	195.0 (162.0–226.3)	<.001
Prothrombin time (s), mean ± SD	13.2 ± 2.1	13.3 ± 2.1	13.0 ± 2.2	.393
INR, mean ± SD	1.1 ± 0.2	1.2 ± 0.2	1.0 ± 0.1	<.001
Autoantibodies [n (%)]				
AMA	144 (64.0)	93 (73.2)	51 (52.0)	.001
AMA-M2	144 (64.0)	94 (74.0)	50 (51.0)	<.001
M2-3E	161 (71.6)	102 (80.3)	59 (60.2)	.001
Sp100	34 (15.1)	15 (11.8)	19 (19.4)	.116
gp210	74 (32.9)	49 (38.6)	25 (25.5)	.039

AMA = antimitochondrial antibody, INR = international normalized ratio, IQR = interquartile range.

Table 2 compares the positivity rates of different AMA detection methods in PBC patients. Of the 225 patients, 111 (49.3%) tested positivity for all 3 AMA detection methods. There was a higher positivity rate for all AMA detection methods in cirrhosis patients compared to non-cirrhosis patients (62.2% vs. 32.7%). AMA-M2 positive patients were further classified as positive or strongly positive based on signal intensity, as were M2-3E positive patients. Of the 144 AMA-M2 positive patients, 107 (74.3%) were strongly positive, including 78 with cirrhosis and 29 without. Of the 161 M2-3E positive patients, 135 (83.9%) were strongly positive, including 91 with cirrhosis and 44 without. Among the 111 patients who tested positive for all AMA detection methods, 96 (86.5%) were strongly positive for AMA-M2 and 103 (92.8%) were strongly positive for M2-3E. The agreement among AMA assays was also assessed. Substantial agreement was found between IIF-AMA and M2-3E (LIA) assay (kappa value = 0.727). Moderate agreement was shown between AMA-M2 (LIA) and M2-3E (LIA) assay (kappa value = 0.505). Fair agreement was found between IIF-AMA and AMA-M2 (LIA) assay (kappa value = 0.363).

**Table 2:** Comparison of positivity by AMA detection methods in cirrhosis and non-cirrhosis patients.

IIF-AMA	AMA-M2 (LIA)	M2-3E (LIA)	Total (n = 225) [n (%)]	Cirrhosis (n = 127) [n (%)]	Non-cirrhosis (n = 98) [n (%)]
-	-	-	43 (19.1)	18 (14.2)	25 (25.5)
**+**	-	-	5 (2.2)	2 (1.6)	3 (3.1)
-	**+**	-	16 (7.1)	5 (3.9)	11 (11.2)
-	-	**+**	5 (2.2)	1 (0.8)	4 (4.1)
**+**	-	**+**	28 (12.4)	12 (9.4)	16 (16.3)
-	**+**	**+**	17 (7.6)	10 (7.9)	7 (7.1)
**+**	**+**	**+**	111 (49.3)	79 (62.2)	32 (32.7)

AMA = antimitochondrial antibody, IIF = indirect immunofluorescence, LIA = line immunoassay.

The associations between autoantibodies and the occurrence of cirrhosis in PBC patients were analyzed using binary logistic regression analysis, and the results are presented in Table 3. In the univariate logistic regression analysis, IIF-AMA, AMA-M2, M2-3E, and gp210 were significantly associated with a higher liver cirrhosis rate. After adjusting for age, gender, T.Bil, and ALP, it was found that neither Sp100 nor gp210 were related to liver cirrhosis. After adjusting for age, gender, T.Bil, ALP, Sp100, and gp210, IIF-AMA (OR: 3.05, 95% CI: 1.59–5.87), AMA-M2 (OR: 3.11, 95% CI: 1.61–6.01), and M2-3E (OR: 3.29, 95% CI: 1.63–6.66) remained significantly associated with an increased liver cirrhosis incidence. Compared to AMA-M2 negative, only a strongly positive AMA-M2 was found to be associated with liver cirrhosis (OR: 3.95, 95% CI: 1.96–7.96). Likewise, only a strongly positive M2-3E was correlated to liver cirrhosis (OR: 4.33, 95% CI: 2.06–9.13).

**Table 3:** Binary logistic regression analysis for cirrhosis.

Variables	Crude model		Model 1		Model 2		Model 3	
	OR (95% CI)	*P*	OR (95% CI)	*P*	OR (95% CI)	*P*	OR (95% CI)	*P*
AMA	2.52 (1.44–4.40)	.001	2.98 (1.61–5.53)	.001	3.02 (1.58–5.80)	.001	3.05 (1.59–5.87)	.001
AMA-M2	2.74 (1.56–4.79)	<.001	3.18 (1.72–5.90)	<.001	3.14 (1.65–5.97)	<.001	3.11 (1.61–6.01)	.001
AMA-M2^a^		<.001		<.001		<.001		<.001
Negative	Reference	-	Reference	-	Reference	-	Reference	-
Positive	1.11 (0.50–2.44)	.798	1.31 (0.55–3.16)	.545	1.48 (0.60–3.67)	.399	1.51 (0.60–3.81)	.386
Strong positive	3.91 (2.12–7.24)	<.001	4.26 (2.20–8.26)	<.001	4.04 (2.03–8.02)	<.001	3.95 (1.96–7.96)	<.001
M2-3E	2.70 (1.49–4.89)	.001	3.07 (1.59–5.92)	.001	3.38 (1.67–6.83)	.001	3.29 (1.63–6.66)	.001
M2-3E^a^		<.001		<.001		<.001		<.001
Negative	Reference	-	Reference	-	Reference	-	Reference	-
Positive	1.14 (0.45–2.89)	.776	1.06 (0.39–2.89)	.905	1.08 (0.37–3.14)	.889	1.08 (0.37–3.13)	.893
Strong positive	3.23 (1.74–5.98)	<.001	3.93 (1.96–7.87)	<.001	4.46 (2.12–9.37)	<.001	4.33 (2.06–9.13)	<.001
Sp100	0.58 (0.27–1.16)	.119	0.64 (0.29–1.43)	.278	0.74 (0.32–1.69)	.473	-	-
gp210	1.83 (1.03–3.27)	.04	1.80 (0.97–3.33)	.061	1.49 (0.77–2.86)	.236	-	-

AMA = antimitochondrial antibody, CI = confidence interval, OR = odds ratio.

Model 1: Adjusted for age and gender.

Model 2: Adjusted for age, gender, total bilirubin, and alkaline phosphatase.

Model 3: Adjusted for age, gender, total bilirubin, alkaline phosphatase, Sp100, and gp210.

a Used as design variable included in logistic regression model.

Table 4 displays the associations between AMA assays and different stages of liver cirrhosis in PBC patients, as determined by multinomial logistic regression analysis. After adjusting for age, gender, T.Bil, and ALP, IIF-AMA (compensated cirrhosis, OR: 3.55, 95% CI: 1.49–8.44; decompensated cirrhosis, OR: 2.86, 95% CI: 1.32–6.18), AMA-M2 (compensated cirrhosis, OR: 4.74, 95% CI: 1.94–11.58; decompensated cirrhosis, OR: 2.51, 95% CI: 1.19–5.33), and M2-3E (compensated cirrhosis, OR: 4.92, 95% CI: 1.74–13.96; decompensated cirrhosis, OR: 2.91, 95% CI: 1.28–6.64) were all found to be associated with different stages of liver cirrhosis. Furthermore, only a strongly positive AMA-M2 and M2-3E were found to be associated with compensated and decompensated cirrhosis.

**Table 4:** Odds ratios of compensated and decompensated cirrhosis compared with non-cirrhosis group using multinomial logistic regression model.

Variables	Compensated cirrhosis (n = 45)				Decompensated cirrhosis (n = 82)			
	Crude model OR (95% CI)	*P*	Model 1 OR (95% CI)	*P*	Crude Model OR (95% CI)	*P*	Model 1 OR (95% CI)	*P*
AMA	3.23 (1.44–7.23)	.004	3.55 (1.49–8.44)	.004	2.23 (1.20–4.14)	.011	2.86 (1.32–6.18)	.007
AMA-M2	3.84 (1.67–8.81)	.002	4.74 (1.94–11.58)	.001	2.32 (1.25–4.31)	.008	2.51 (1.19–5.33)	.016
AMA-M2^a^								
negative	Reference		Reference		Reference		reference	
positive	1.52 (0.48–4.83)	.474	2.05 (0.60–6.94)	.251	0.95 (0.39–2.34)	.915	1.24 (0.42–3.67)	.702
strong positive	5.52 (2.30–13.25)	<.001	6.33 (2.49–16.12)	<.001	3.31 (1.69–6.49)	<.001	3.19 (1.44–7.09)	.004
M2-3E	4.30 (1.66–11.11)	.003	4.92 (1.74–13.96)	.003	2.19 (1.14–4.21)	.019	2.91 (1.28–6.64)	.011
M2-3E^a^								
negative	Reference		Reference		Reference		reference	
positive	2.17 (0.57–8.17)	.254	2.10 (0.52–8.57)	.300	0.82 (0.28–2.45)	.724	0.60 (0.15–2.46)	.480
strong positive	5.02 (1.91–13.24)	.001	6.10 (2.09–17.80)	.001	2.66 (1.35–5.22)	.005	4.11 (1.72–9.82)	.001

AMA = antimitochondrial antibody, CI = confidence interval, OR = odds ratio.

Model 1: Adjusted for age, gender, total bilirubin, and alkaline phosphatase.

a Used as design variable included in logistic regression model.

## 4. Discussion

In this retrospective study, we enrolled 225 patients with PBC to investigate the association between AMA and the occurrence of liver cirrhosis in PBC patients. The AMA assays used in this study included IIF-AMA, AMA-M2 (LIA), and M2-3E (LIA). The results show that IIF-AMA, AMA-M2, and M2-3E were all significantly associated with the development of cirrhosis in PBC patients, regardless of multivariable adjustment by performing multivariate logistic regression analysis. Furthermore, significant associations between AMA assays and different stages of cirrhosis were also found.

Traditionally, AMA is used only as a serological marker for the diagnosis of PBC and is reported to not associate with disease severity or prognosis in PBC patients.^[7]^ However, emerging evidence suggests that AMA titers may be directly or indirectly related to the outcome of PBC patients. In a large-scale characterization study, the mortality rate of AMA-positive patients without PBC was elevated regardless of the risk of developing PBC.^[8]^ Moreover, increased AMA IgG and IgA titers during follow-up were associated with biochemically and/or histologically advanced disease in patients with PBC.^[9]^ UDCA is recommended as first-line therapy for PBC due to its ability to dramatically alter the natural course of the disease and delay histological progression.^[13]^ PBC patients who exhibit complete biochemical responses to UDCA have better outcomes than non-responders.^[14]^ In a 28-year cohort study, UDCA responders exhibited decreased AMA IgG titers at 1 year after treatment, which persisted until the last follow-up.^[10]^ Our study also found that AMA was independently associated with the development of cirrhosis in PBC patients. Additionally, higher AMA titers were linked to an increased risk of developing liver cirrhosis.

IIF method is the most widely accepted for detecting AMA. At least 9 subtypes of AMA exist, and 4 of them (M2, M4, M8, and M9) have been reported to be related to PBC.^[15]^ The most specific subtype for PBC is the M2 subtype of AMA (AMA-M2), which is routinely detected in clinical laboratories. AMA-M2 mainly targets PDC-E2, BCOADC-E2, and OGDC-E2. In this study, AMA-M2 (native M2) and M2-3E (BPO) were detected using LIA. The sensitivity of pooled AMA using IIF and LIA was found to be 80.9%, comparable to the results of a meta-analysis conducted by Hu et.al.^[16]^ There was the highest agreement between IIF-AMA and M2-3E (LIA). Although different AMA testing methods were used in this study, the results consistently showed a significant association between AMA or AMA-M2 and the development of liver cirrhosis in PBC patients.

The pathogenesis of PBC involves the immune system recognizing biliary epithelial cells as foreign and attacking them. Evidence suggests that a breakdown of tolerance against biliary epithelial cells may be due to a combination of genetic and environmental factors.^[3]^ This process, called cholangitis, can lead to cirrhosis of the liver. Notably, the triad of PBC monocytes, biliary apoptosis, and AMA can lead to an intense proinflammatory cytokine burst.^[17]^ However, a direct association between AMA titer and the progression of PBC disease has not been found. Further research is needed to investigate whether AMA is directly related to PBC disease progression and its possible underlying mechanisms.

PBC-specific ANA exhibit 2 distinct immunofluorescence patterns: the multiple nuclear dots pattern (including Sp100) and the rim-like/membranous pattern (including gp210). These patterns can help diagnose PBC, regardless of AMA status.^[5,18]^ Approximately 20% of PBC patients have been reported to have antibodies targeting Sp100 and gp210, which is consistent with our research findings.^[19]^ Additionally, several studies have investigated the relationship between anti-Sp100 and anti-gp210 antibodies and the prognosis of PBC patients.^[6,20,21]^ In our study, we did not find an association between anti-Sp100 and the prognosis of PBC patients, which aligns with previous research.^[6,20]^ The presence of anti-gp210 antibody has been linked to 2 distinct types of disease progression in PBC: hepatic failure and portal hypertension.^[6]^ In our study, we observed an association between anti-gp210 antibody and liver cirrhosis in the univariate analysis. However, this association did not hold in the multivariate analysis. Further validation is needed to confirm these observations.

Various criteria have been proposed to assess biochemical responses to UDCA, including Paris-I, Paris-II, GLOBE score and so on.^[22–24]^ Among them, ALP and T.Bil are the most frequently used biochemical indicators. In the current study, to reduce the impact of UDCA response on the outcome, we adjusted for ALP and T.Bil using logistic regression. The results showed that the association of AMA and the occurrence of cirrhosis remained robust after adjusting ALP and T.Bil. Furthermore, age and sex have been found to affect the long-term outcome of PBC patients. Young age at onset predicts poor prognosis for PBC, and elderly patients have a high risk of the occurrence of hepatocellular carcinoma.^[25,26]^ Male sex is related to poor UDCA response and a high risk of the development of hepatocellular carcinoma.^[26,27]^ Therefore, we also adjusted for age and sex by logistic regression, and the association between AMA and cirrhosis remained robust.

The present study has some limitations. Firstly, this was a retrospective single-center design, which may limit the generalizability of the findings to other settings. Secondly, although UDCA response has been found to be associated with prognosis in PBC patients, routine evaluation was not conducted for each patient in the study. This may affect the results, despite adjusting for relevant factors through multivariate analysis. Thirdly, the diagnosis of cirrhosis may have been suboptimal in some patients due to the lack of liver biopsies. Additionally, it is important to consider other factors that may impact prognosis in PBC patients, such as comorbidities and lifestyle factors, which were not fully assessed in the present study. Therefore, prospective large-scale studies are needed to further verify the results.

## 5. Conclusions

Our findings suggest that AMA is dependently associated with the occurrence of liver cirrhosis in PBC patients. Moreover, AMA is also related to different stages of liver cirrhosis, including compensated and decompensated cirrhosis. However, additional large-scale and well-designed prospective studies are necessary to verify our results.

## Author contributions

**Conceptualization:** Aifang Xu.

**Formal analysis:** Yujiao Jin, Miaochan Wang.

**Investigation:** Miaochan Wang, Yuan Liu.

**Methodology:** Yujiao Jin, Miaochan Wang, Yuan Liu.

**Resources:** Aifang Xu.

**Supervision:** Aifang Xu.

**Validation:** Yuan Liu, Aifang Xu.

**Writing – original draft:** Yujiao Jin, Miaochan Wang.

**Writing – review & editing:** Yujiao Jin, Aifang Xu.
